# Hepatoprotective mechanism of *Silybum marianum* on nonalcoholic fatty liver disease based on network pharmacology and experimental verification

**DOI:** 10.1080/21655979.2022.2037374

**Published:** 2022-02-16

**Authors:** Guoyan Jiang, Chunhong Sun, Xiaodong Wang, Jie Mei, Chen Li, Honghong Zhan, Yixuan Liao, Yongjun Zhu, Jingxin Mao

**Affiliations:** a Department of Emergency, The Third Affiliated Hospital of Chongqing Medical University, Chongqing, China; b Chongqing Medical and Pharmaceutical College, School of Clinical medicine, Chongqing, China; c Department of periodontal, Stomatological Hospital of Chongqing Medical University, Chongqing, China; d Department of Biology, Chemistry, Pharmacy, Free University of Berlin, Berlin, Germany; e College of Pharmaceutical Sciences, Southwest University, Chongqing, China; f Department of Orthopedics, The Ninth People’s Hospital of Chongqing, Chongqing, China; g College of Basic Medical Science, Southwest University, Chongqing, China

**Keywords:** Nonalcoholic fatty liver disease, NAFLD, *Silybum marianum*, silymarin, network pharmacology, hepatoprotective mechanism

## Abstract

The study aimed to identify the key active components in *Silybum marianum* (*S. marianum*) and determine how they protect against nonalcoholic fatty liver disease (NAFLD). TCMSP, DisGeNET, UniProt databases, and Venny 2.1 software were used to identify 11 primary active components, 92 candidate gene targets, and 30 core hepatoprotective gene targets in this investigation, respectively. The PPI network was built using a string database and Cytoscape 3.7.2. The KEGG pathway and GO biological process enrichment, biological annotation, as well as the identified hepatoprotective core gene targets were analyzed using the Metascape database. The effect of silymarin on NAFLD was determined using H&E on pathological alterations in liver tissues. The levels of liver function were assessed using biochemical tests. Western blot experiments were used to observe the proteins that were expressed in the associated signaling pathways on the hepatoprotective effect, which the previous network pharmacology predicted. According to the KEGG enrichment study, there are 35 hepatoprotective signaling pathways. GO enrichment analysis revealed that 61 biological processes related to the hepatoprotective effect of *S. marianum* were identified, which mainly involved in response to regulation of biological process and immune system process. Silymarin was the major ingredient derived from *S. marianum,* which exhibited the hepatoprotective effect by reducing the levels of ALT, AST, TC, TG, HDL-C, LDL-C, decreasing protein expressions of IL-6, MAPK1, Caspase 3, p53, VEGFA, increasing protein expression of AKT1. The present study provided new sights and a possible explanation for the molecular mechanisms of *S. marianum* against NAFLD.

## GRAPHICAL ABSTRACT

**Figure:**
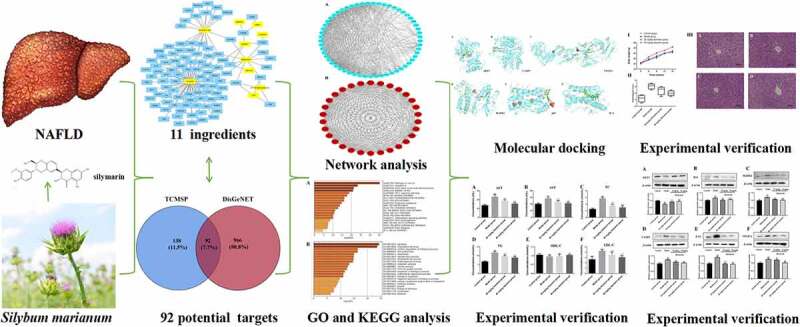

## 1. Introduction

Nonalcoholic fatty liver disease (NAFLD) is a metabolic stress liver damage that includes nonalcoholic fatty liver, nonalcoholic steatohepatitis, hepatic cirrhosis, and hepatocellular carcinoma [1,2]. NAFLD is not only linked to an increased risk of liver illness, disability, and death but it is also linked to an increased risk of metabolic syndrome (MetS), type 2 diabetes, and cancer-related diseases [3]. NAFLD has become the most frequent chronic liver disease, owing to the rising prevalence of obesity and MetS, with the leading source of aberrant liver biochemical indicators in health examinations [4]. Furthermore, an increasing number of persons with chronic hepatitis B virus infection are co-infected with NAFLD, putting people’s lives and health at risk [5]. In China, the prevalence of NAFLD has risen dramatically in the last 10 years. Previous research indicated that the total number of NAFLD cases would rise by 0% to 30%, and the prevalence of NASH would rise by 15% to 56% between 2016 and 2030 [6]. NAFLD has already surpassed hepatitis C as the primary cause of chronic liver disease in China [7] since the aging population, liver mortality, and advanced liver disease have all increased dramatically. Imaging or histological evidence of diffuse hepatocyte steatosis, as well as the exclusion of alternative causes of hepatic steatoses, such as alcohol consumption, are used to diagnose NAFLD [8].

Because there are no identifiable symptoms or indications, most individuals are suspected of having NAFLD based on the inadvertent discovery of elevated serum alanine aminotransferase (ALT) and γ-glutamyl transpeptidase (GGT) levels or imaging data demonstrating diffuse fatty liver [9]. NAFLD is evaluated by quantifying the degree of liver steatosis and fibrosis, determining whether there are metabolic and cardiovascular risk factors and complications, determining whether there is liver inflammation damage, and determining whether it is associated with other causes of liver disease [10]. NAFLD treatment includes exercise (at least 150 min per week of brisk walking); diet control (reducing current diet by 500 kcal/d); weight control (for overweight and obese patients, reduce 5–10% of body weight within the first 6 months); and avoidance or use of drugs with potential liver toxicity (such as acetaminophen, rifampicin, cimetidine, tetracycline, and others) [11–13]. Medications include hepatoprotective drugs, anti-inflammatory drugs, insulin sensitizers drugs, and hypoglycemic drugs [14].

For a long time, herbs and herbal therapies have been used to treat relevant liver illnesses, including NAFLD [15]. Milk thistle (*Silybum marianum* L.) is a herbaceous plant of the genus Silybum in the Compositae family that originates in Southern Europe and North Africa [16]. It has been reported that *Silybum marianum* L. (*S. marianum*) was used to treat liver and gallbladder diseases in the Western world as early as the fourth century BC. It was also revealed that *S. marianum* exhibits anti-inflammatory, immunomodulating, antifibrotic, antioxidant, and liver-regenerating properties in patients with alcoholic liver disease, nonalcoholic fatty liver disease, viral hepatitis, and drug-induced liver injury [17]. Silymarin is a mixture of lipophilic and flavonoid lignans extracted directly from dried seeds of *S. marianum*, which mainly includes isosilychristin, isosilybin, silybin, silychristin, silydianin, and toxifolin [18]. It was reported that silymarin exhibits pharmacological effects on lowering blood lipids, antioxidant, preventing diabetes, anti-inflammatory, inhibiting various tumors, neuroprotection, and immune regulation [19]. In addition, silymarin also exhibits various pharmacological activities, which can protect liver cell membranes, prevent liver cell degradation, promote liver purification, and help liver detoxification [20].

The concept of ‘multi-target and multi-component treatments’ is emphasized in network pharmacology, which has the benefits of total treatment and may overcome the shortcomings of ‘single target.’ *S. marianum*’s pharmacological properties have been investigated for many years, and it is widely used in clinical applications due to its good curative impact, few side effects, easy oral administration, and low cost [21]. However, due to the complex composition of *S. marianum*, its primary material foundation (including significant active components/ingredients) and molecular mechanism for treating NAFLD have yet to be completely investigated. Furthermore, no relevant network pharmacology was used to investigate the effects of *S. marianum* active components on the mechanism of NAFLD treatment. As a result, the network pharmacology technique was utilized in conjunction with a relevant database to screen the primary active components in *S. marianum* and extensively investigate its protective effect on NAFLD targets, related signaling cascades, and biological processes. Furthermore, experimental validation based on network pharmacology analysis was carried out to elaborate and confirm the targets and related signaling pathways in NAFLD. The key active components in *S. marianum* and hepatoprotective mechanism against NAFLD are still unclear. The present study aimed to figure out the active hepatoprotective ingredient of *S. marianum* and study the specific mechanism of NAFLD.

## 2. Materials and methods

### 2.1. The network database and software used in analysis and plotting

UniProt Protein database (https://www.uniprot.org/) [22], DisGeNET database (https://www.disgenet.org/) [23], String database (https://string-db.org/) [24], Metascape database (https://metascape.org/gp/index.html#/main/step1) [25], TCM pharmacology database (TCMSP, https://tcmspw.com/tcmsp.php/) [26], and software Cytoscape 3.7.2 [27] were utilized for analysis and plotting, respectively.

### 2.2. *Ascertained the active ingredients and targets of* S. marianum

The active ingredients/components and targets of *S. marianum* were obtained by searching ‘*Silybum marianum*’ on the TCMSP database platform. According to the standard of oral bioavailability (OB) ≥ 30%, drug-likeness (DL) ≥ 0.18, and molecular weight (MW) < 500, the main active ingredients/components and targets of *S. marianum* were ascertainedy (Table 1).

**Table 1.** Easily absorbed active components and their basic parameters in *S. marianum.*

Mol ID	Molecule Name	MW	AlogP	Hdon	Hacc	OB (%)	DL
MOL001439	arachidonic acid	304.52	6.41	1	2	45.57	0.2
MOL001736	(-)-taxifolin	304.27	1.49	5	7	60.51	0.27
MOL000449	stigmasterol	412.77	7.64	1	1	43.83	0.76
MOL007180	Vitamin E	490.69	3.78	3	9	32.29	0.7
MOL007449	24-methylidenelophenol	412.77	7.75	1	1	44.19	0.75
MOL007450	silybin	482.47	2.62	5	10	0.93	0.93
MOL007451	silydianin	482.47	0.95	5	10	59.65	0.76
MOL007454	silymonin	466.47	1.5	4	9	81.81	0.8
MOL007455	silandrin	466.47	3.17	4	9	64.14	0.94
MOL000098	quercetin	302.25	1.50	5	7	46.43	0.28
MOL000953	CLR	386.73	7.38	1	1	37.87	0.68

**Table 2.** Hepatoprotective on NAFLD targets of active components from *S. marianum*

Target name	Gene
Prostaglandin G/H synthase 1	PTGS1
Sodium-dependent noradrenaline transporter	SLC6A2
Transcription factor p65	RELA
Mitogen-activated protein kinase 1	MAPK1
Pro-epidermal growth factor	EGF
Cyclin-dependent kinase 4	CDK4
Caspase-3	CASP3
Peroxisome proliferator-activated receptor gamma	PPARG
Tumor necrosis factor receptor superfamily member 1A	TNFRSF1A
Protein kinase C beta type	PRKCB
Nitric oxide synthase, endothelial	NOS3
Arachidonate 5-lipoxygenase	ALOX5
Selenoprotein P	SELP
Beta-galactosidase	GLB1
Aldehyde dehydrogenase, mitochondrial	ALDH2
ATP-binding cassette transporter A1	ABCA1
Mitochondrial uncoupling protein 2	UCP2
Cholesteryl ester transfer protein	CETP
ATP-binding cassette sub-family G member 1	ABCG1
Tumor necrosis factor receptor superfamily member 1B	TNFRSF1B
Heat shock protein HSP 90-alpha	HSP90AA1
Mineralocorticoid receptor 3	NR3C2
Nuclear receptor coactivator 2	NCOA2
Beta-2 adrenergic receptor	ADRB2
Aldo-keto reductase family 1 member B1	AKR1B1
Muscarinic acetylcholine receptor	CHRM3
5-hydroxytryptamine (Serotonin) receptor 2A	HTR2A
Androgen receptor	AR
Dipeptidyl peptidase 4	DPP4
Epidermal growth factor receptor	EGFR
RAC-alpha serine/threonine-protein kinase	AKT1
Vascular endothelial growth factor A	VEGFA
BCL2 protein	BCL2
Proto-oncogene c-Fos	FOS
Bax protein	BAX
Matrix metallopeptidase −2	MMP2
Matrix metalloproteinase-9	MMP9
Interleukin-10	IL10
Transcription factor AP-1	JUN
Interleukin 6	IL6
Activator of 90 kDa heat shock protein ATPase homolog 1	AHSA1
Cellular tumor antigen p53	TP53
Caspase-8	CASP8
Superoxide dismutase 1	SOD1
Protein kinase C	PRKCA
Matrix metalloproteinase 1	MMP1
Hypoxia-inducible factor 1	HIF1A
Signal transducer and activator of transcription 1	STAT1
Endoplasmic reticulum chaperone BiP	HSPA5
Acetyl-CoA carboxylase 1	ACACA
Heme oxygenase 1	HMOX1
Cytochrome P450 3A4	CYP3A4
Cytochrome P450 1A2	CYP1A2
Caveolin	CAV1
Myc proto-oncogene protein	MYC
Cytochrome P450 1A1	CYP1A1
Interleukin-1 beta	IL1B
C-C motif chemokine 2	CCL2
Vascular cell adhesion protein 1	VCAM1
Multifunctional fusion protein	CXCL8
Dual oxidase 2	DUOX2
Transforming growth factor beta	TGFB1
Sulfotransferase	SULT1E1
Interleukin-2	IL2
Orphan nuclear receptor PXR	NR1I2
Serpin peptidase inhibitor	SERPINE1
Type I collagen alpha 1(I) chain	COL1A1
Interferon gamma	IFNG
Interleukin-1 alpha	IL1A
Myeloperoxidase	MPO
Neutrophil cytosol factor 1	NCF1
Glutathione S-transferase P	GSTP1
Nuclear factor erythroid 2-related factor 2	NFE2L2
NAD(P)H dehydrogenase [quinone] 1	NQO1
Poly polymerase	PARP1
Aryl hydrocarbon receptor	AHR
Solute carrier family 2, facilitated glucose transporter member 4	SLC2A4
Collagen alpha-1(III) chain	COL3A1
Constitutive androstane receptor	NR1I3
Insulin receptor	INSR
Peroxisome proliferator-activated receptor alpha	PPARA
Peroxisome proliferative activated receptor	PPARD
C-reactive protein	CRP
C-X-C motif chemokine 10	CXCL10
Inhibitor of nuclear factor kappa-B kinase subunit alpha	CHUK
Secreted phosphoprotein 1	SPP1
Runt-related transcription factor	RUNX2
Cathepsin D isoform 2	CTSD
Insulin-like growth factor 2	IGF2
Paraoxonase 1	PON1
Glutathione S-transferase Mu 1	GSTM1
Glutathione S-transferase	GSTM2

### 2.3 *Built the target network of active components of* S. marianum

From ‘Section **2.2**’ and uniform conversion to shortened gene names, the UniProt database was used to identify the active components and related target proteins of *S. marianum*. The active components of *S. marianum* and targets were imported into Cytoscape 3.7.2 for data construction, visualization, and analysis. The network graph of ‘active components–targets of *S. marianum*’ was built, and topological data such as degree value and betweenness were calculated. The topological features of *S. marianum* were investigated using the ‘network analyzer’ tool. The betweenness centrality (BC) of nodes in large complex pharmacological networks is investigated.

### 2.4. *Collection of* S. marianum *active compounds’ protective effects on NAFLD-related targets*

‘Nonalcoholic fatty liver disease or NAFLD,’ was utilized as a keyword to search the DisGeNET database for target information relating to NAFLD’s protective effect. The NAFLD target and *S. marianum*’s active ingredient targets were mapped using Venny 2.1, as well as the common target was chosen as the relevant target of *S. marianum*’s active ingredient against NAFLD. The target network of ‘active compound-hepatoprotective on NAFLD of *S. marianum*’ was then constructed in Cytoscape 3.7.2 software, with its topological properties were studied using the ‘network analyzer’ function.

### 2.5. Development of a target protein–protein interaction network

Using a string database, the target protein–protein interaction (PPI) network of related target proteins was built. The species (protein species) was set to ‘Homo sapiens,’ and the minimal interaction threshold (0.7) was set to ‘medium confidence.’ The remaining parameters were left at their default values. The network analyzer function in Cytoscape 3.7.2 software is then used to investigate the topological properties of the PPI network. After the process, the PPI network diagram was created.

### 2.6. Analysis of KEGG pathways and GO biological process enrichment

To enable full biological function annotation information, the Kyoto Encyclopedia of Genes and Genomes (KEGG) and gene function annotation (GO) were used in the study. The Metascape database performed a KEGG pathway and GO biological process enrichment analysis on core genes obtained in Section **2.5**. All of the core genes were tested, with the critical value of significant functions and pathways set at the threshold *P* < 0.05. The primary routes and biological processes via which the main active components of *S. marianum* exert considerable hepatoprotective pharmacological effects in NAFLD were identified. Furthermore, the effects of *S. marianum* on NAFLD have been established. To validate the liver protection mechanism of *S. marianum* against NAFLD across numerous targets and multiple pathways, the KEGG mapper function was utilized to mark the target of the selected active ingredient on the pathway associated with NAFLD.

### 2.7. Experimental verification

#### 2.7.1. Materials and reagents

SPF male C57BL/6 J mice aged 7–8 weeks and weighing 20–22 g were obtained from the Chongqing Academy of Traditional Chinese Medicine. Nanjing Jiancheng Bioengineering Institute (Nanjing, China) provided diagnostic kits for the determination of alanine aminotransferase (ALT), aspartate aminotransferase (AST), triglyceride (TG), total cholesterol (TC), high-density lipoprotein (HDL-C), and low-density lipoprotein (LDL-C). Proteintech Co., Ltd (Wuhan, China) provided rabbit anti-serine/threonine protein kinase 1 (AKT 1), interleukin 6 (IL-6), mitogen-activated protein kinase 1 (MAPK1), cysteinyl aspartate specific proteinase (Caspase 3), p53, TNF-polyclonal primary antibodies from mice, an anti-mouseIgG-HRP-conjugated secondary antibody from goat, and anti-rabbit IgGHRP-conjugated secondary antibody goat. Sangon Biotech Co. Ltd (Shanghai, China) provided the total protein extraction kit. Chongqing Yonghui Supermarket (Chongqing, China) supplied sucrose, lard, and eggs. Dalian Beier Pharmaceutical Co., Ltd (Dalian, China) provided cholesterol, sodium cholate, and propylthiouracil.

#### 2.7.2. Animals and experimental protocols

After the experimental mice had been adaptively fed for 7 days in a row, they were randomly separated into four groups: control, model, low-dose silymarin, and high-dose silymarin, with 20 mice in each group. All mice were housed in a specific compartment with controlled air conditions and were free to eat and drink during the experiment.

- Control group: mice were given intragastric PBS for consecutive 16 weeks.
- Model group: mice were given an intragastric nutritional solution (the formula was 10% sucrose, 10% lard, 3% egg yolk, 2% cholesterol, 1% sodium cholate, and 0.5% propylthiouracil) for consecutive 16 weeks.
- Low-dose silymarin group (20 mg/kg silymarin group): For consecutive 16 weeks, mice were given silymarin at a level of 20 mg/kg body weight per day along with a nutritional solution.
- High-dose silymarin group (40 mg/kg silymarin group): For consecutive 16 weeks, mice were treated with silymarin at a level of 40 mg/kg body weight/day and nutritional solution.

#### 2.7.3. Molecular docking between silymarin and NAFLD-related key targets

Molecular docking of known key targets with silymarin, the docking affinity reflects its stability. Using PDB database [28] and PubChem [29] to download the molecular structure of core protein and the structure of silymarin, respectively. Then, Auto Dock Tools software [30] was used to perform molecular docking processing. The PyMol 2.4.0 software [31] was utilized finally for visualization.

#### 2.7.4. Histopathological examination

After consecutive 16 weeks of feeding, all of the mice were anesthetized with an intraperitoneal injection of 1% sodium pentobarbiturate and sacrificed. For histopathological examination, liver tissues of mice were obtained and fixed in a solution, which contained 10% neutral buffered formalin. For routine pathological examination, a 4 μm thick section was cut and stained with hematoxylin–eosin (H&E). A microscope was used to examine the pathological changes in liver tissues, and photographs were taken with a 200-fold magnifying glass.

#### 2.7.5. Biochemical assays

Mice were subjected to fasting with water for 12 h, and were anesthetized by intraperitoneal injection of 1% sodium pentobarbiturate. Mice eyeballs were removed to collect the blood. The blood was centrifuged at 5,000 r/min for 15 min, at 4°C for 2 h,  centrifuged the serum into a 1.5 mL centrifuge tube. The levels of ALT, AST, TG, TC, HDL-C, and LDL-C were measured, respectively, by HITACHI 7600–020 automatic biochemistry analyzer (Hitachi Company, Japan).

#### 2.7.6. Western blot assays

Western blot assays were used to survey the protein expression of the related signaling pathways predicted by the previous network pharmacology in mice livers. The mice liver tissue lysate was separated using 12.5% SDS-PAGE and transferred to a PVDF membrane before being washed three times with TBST. The blots were then blocked in 5% skim milk for 2 h at 37°C before being incubated overnight at 4°C with the following primary antibodies: AKT1 (1:1000), IL-6 (1:1000), MAPK1 (1:2500), Caspase 3 (1:2500), p53 (1:2500), and VEGFA (1:2500). Secondary IgG-HRP-conjugated antibodies were added and incubated at 37°C for 2 h. Image J software (version 1.8.0) was used to quantify and visualize target proteins, with *β*-actin serving as an internal standard.

### 2.8. Ethics statement

All of the relative animal studies were carried out in accordance with the rules for the care and use of laboratory animals, which were authorized by the Southwest University’s Office of Experimental Animal Management Committee (Ethical number: 2019–14).

### 2.9. Statistical analysis

Using SPSS 19.0 statistical software (SPSS Inc., Chicago, IL, USA) for statistical analysis. The differences between the two groups were compared using independent samples t-tests. Differences were considered significant at *P* < 0.05 or extremely significant at *P* < 0.01, respectively. All statistical analyses were performed using GraphPad Prism 8.0 (La Jolla, California, USA).

## 3. Results

The purpose of the current study was to find out the active hepatoprotective ingredient of *S. marianum* and study the specific mechanism of NAFLD . We applied network pharmacology and experimental validation to explore the pharmacological mechanism of *S. marianum* against NAFLD. The active hepatoprotective ingredient of *S. marianum* against NAFLD was silymarin. It could exhibit the hepatoprotective effect on NAFLD by improving the levels of ALT, AST, TC,TG, HDL-C, LDL-C, decreasing protein expressions of IL-6, MAPK1, Caspase 3, p53, VEGFA, and increasing protein expression of AKT1.

### 3.1. *Screening results of main active chemical components in* S. marianum

TCMSP database screening parameters of OB ≥ 30%, DL ≥ 0.18, and MW < 500 resulted in the discovery of 11 active components of *S. marianum,* which including arachidonic acid, (-)-taxifolin, stigmasterol, Vitamin E, 24-methylidenelophenol, silybin, silydianin, silymonin, silandrin, quercetin, and CLR, respectively.

### 3.2. *Active compounds of* S. marianum *correspond to potential gene targets*

**Figure 1.**
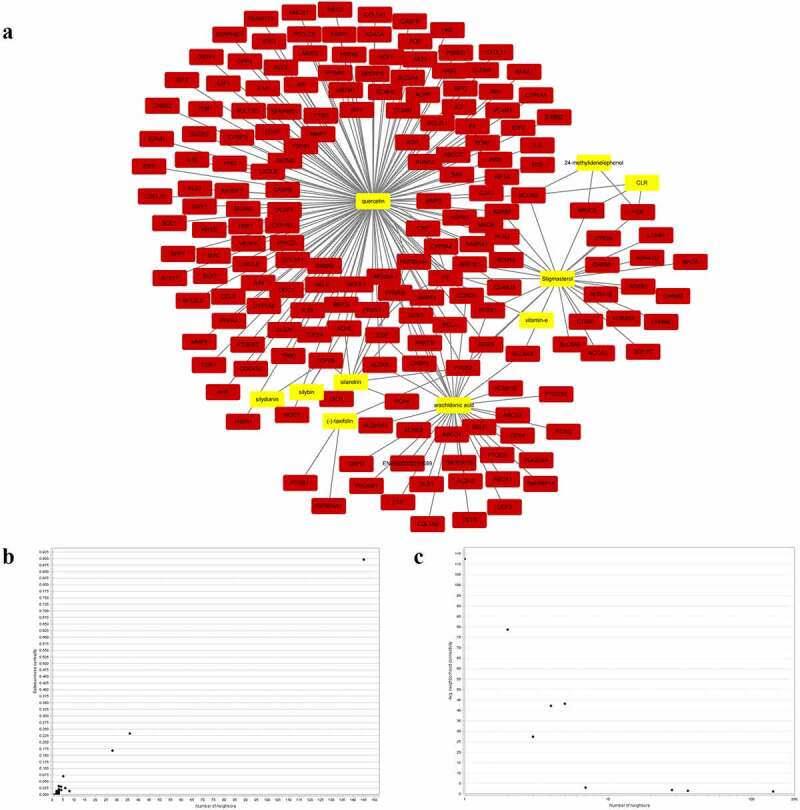
The diagram and distribution map of degree value and betweenness centrality associated with active components of *S. marianum*-prediction target network.

Based on the TCMSP database, a total of 230 putative gene targets matching the 11 active chemicals of *S. marianum* were finally identified. Among them, arachidonic acid has 36 gene targets, (-)-taxifolin has 3 gene targets, stigmasterol has 28 gene targets, Vitamin E has 1 gene target, 24-methylidenelophenol has 3 gene targets, silybin has 2 gene targets, silydianin has 1 gene target, silymonin has 0 gene target, silandrin has 8 gene targets, quercetin has 145 gene targets, and CLR has 3 gene targets. Creating a ‘compound-gene target network’ with Cytoscape 3.7.2 software (Figure 1(a), yellow nodes represent drugs and red nodes represent gene targets). Figure 1(b) and Figure 1(c) showed the overall general distribution of each node’s average degree value in the network diagram, as well as the distribution of betweenness centrality (BC), respectively.

### 3.3. S. marianum *active ingredient-NAFLD target network*

**Figure 2.**
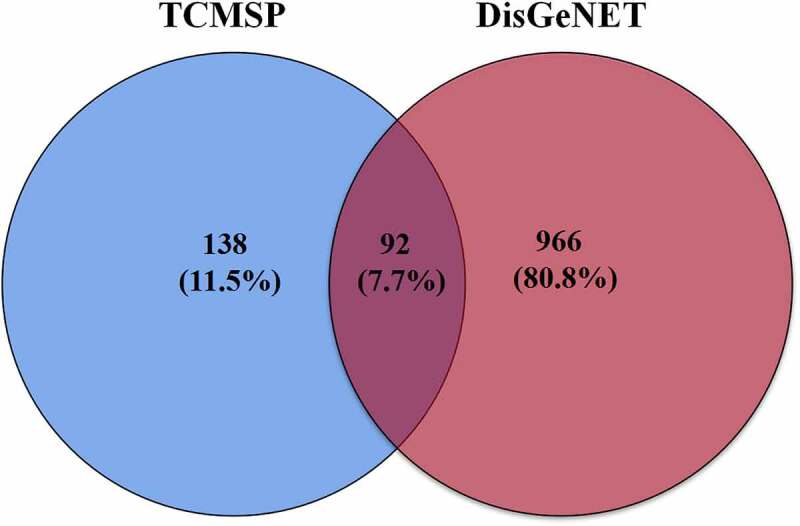
Common targets in Venn diagram.

**Figure 3.**
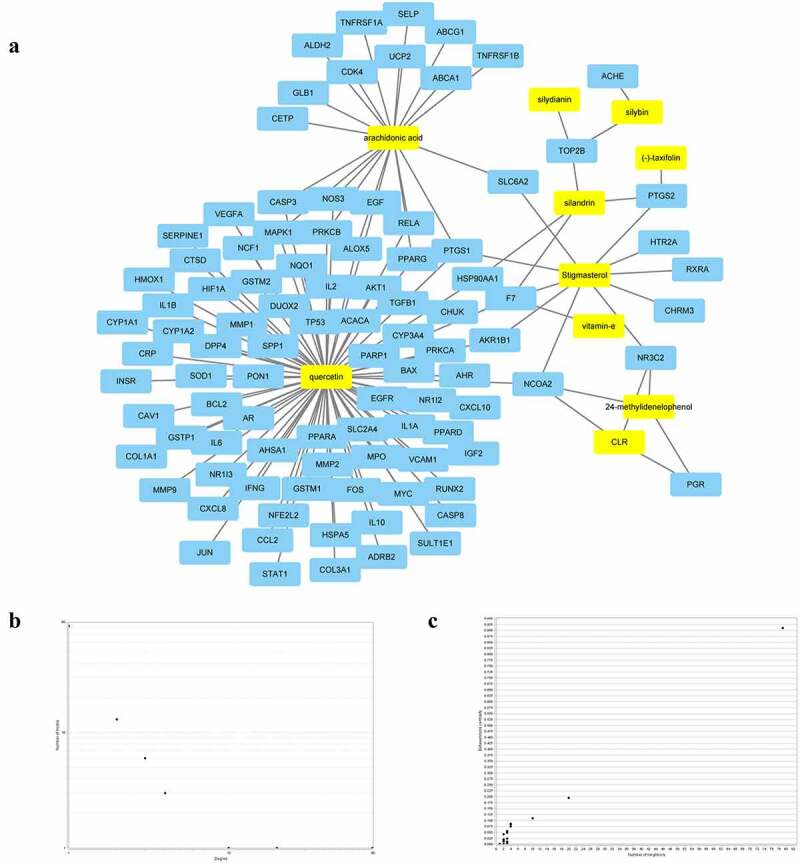
The diagram and distribution map of degree value and betweenness centrality associated with active components of *S. marianum*–hepatoprotective-NAFLD target network.

Using the DisGeNET database, a total of 1,058 targets linked to NAFLD were retrieved. The active compound gene targets from the TCMSP database were mapped to NAFLD-related gene targets from the DisGeNET database. Venny 2.1 was used to analyze and illustrate the Venn diagram, yielding 92 common targets (Figure 2, Table 2). To structure the target network of ‘active compound-hepatoprotective on NAFLD’ of *S. marianum*, all data were imported into Cytoscape 3.7.2 (Figure 3(a), yellow nodes represent drugs and blue nodes represent gene targets). Figure 3(b,c)) show the overall findings of the average degree distribution and the median central distribution of each node in the network diagram.

### 3.4. Analysis of gene target on PPI network

**Figure 4.**
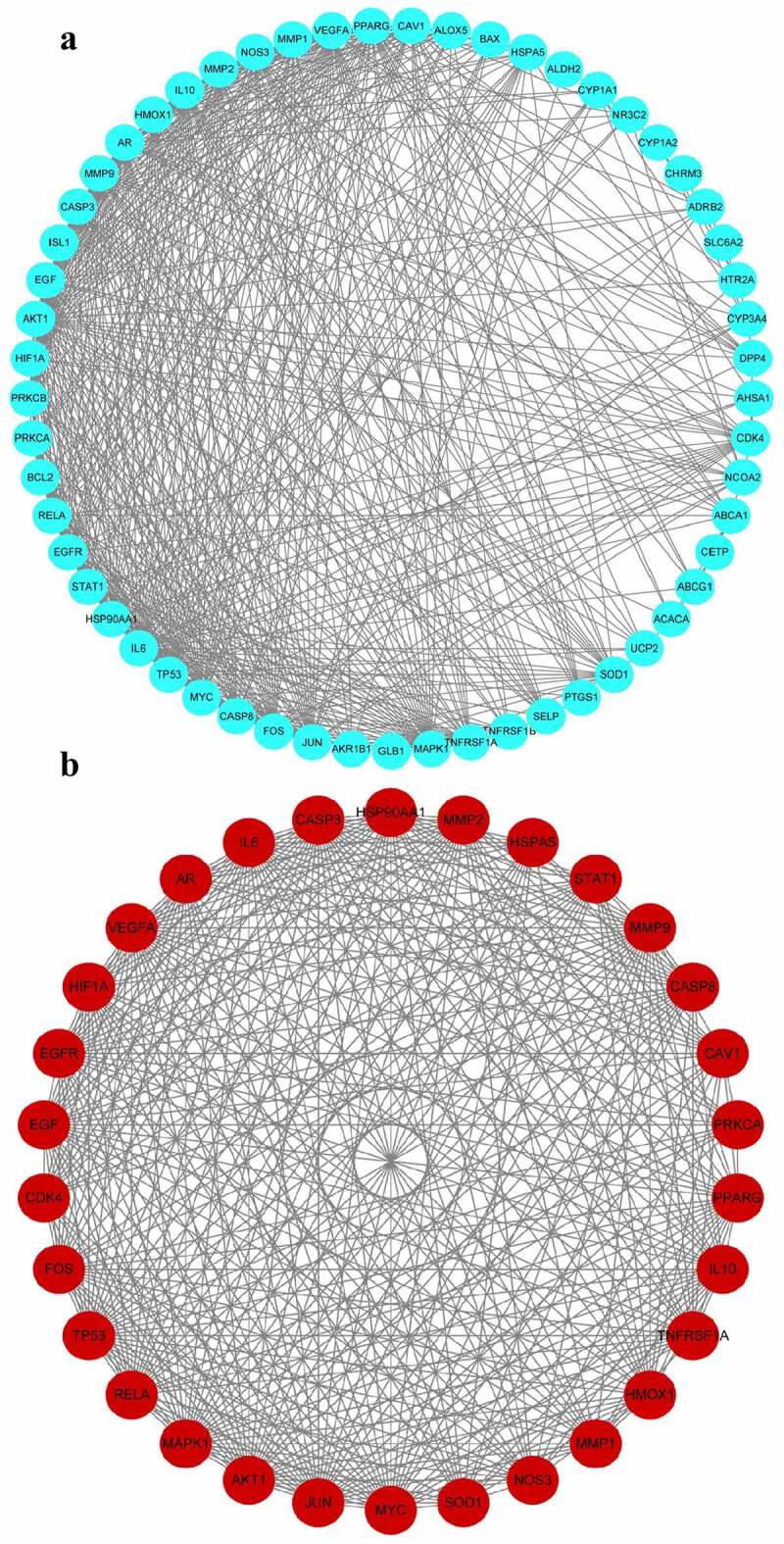
PPI network of active compounds-hepatoprotective-NAFLD target protein of *S. marianum*.

To develop a PPI network of gene targets, the data of hepatoprotective on NAFLD targets of *S. marianum* in Section 3.3 were imported into the string database (version 11.0). Then, for visual analysis, import the PPI network data into Cytoscape 3.7.2 software (Figure 4(a)), set the threshold value of degree > 15, and choose 30 core targets based on the degree value from high to low (Table 3). AKT1, IL6, CASP3, VEGFA, TP53, PPARG, JUN, MAPK1, MMP9, EGFR, EGF, HSP90AA1, IL10, MYC, FOS, NOS3, AR, RELA, MMP2, HMOX1, CAV1, CASP8, HIF1A, TNFRSF1A, STAT1, SOD1, CDK4, MMP1, HSPA5, and PRKCA were among the primary targets. Cancer, hepatitis B, hepatitis C, NAFLD, endocrine resistance, insulin resistance, type 2 diabetes, inflammatory bowel disease, and other disorders are linked to these target proteins. The Cytoscape 3.7.2 software was used to visualize and analyze these primary targets (Figure 4(b)). Finally, the entire PPI network of NAFLD-related target proteins was established.

**Table 3.** Core targets of *S. marianum* on NAFLD and their topological characteristics

Core target name	Gene name	Degree	Betweenness centrality
RAC-alpha serine/threonine-protein kinase	AKT1	43	0.1114036
Interleukin 6	IL6	42	0.07365669
Caspase-3	CASP3	38	0.04502805
Vascular endothelial growth factor A	VEGFA	38	0.03392199
Cellular tumor antigen p53	TP53	37	0.03285972
Peroxisome proliferator-activated receptor gamma	PPARG	33	0.03477514
Transcription factor AP-1	JUN	32	0.01444133
Mitogen-activated protein kinase 1	MAPK1	32	0.03548709
Matrix metalloproteinase-9	MMP9	32	0.01193582
Epidermal growth factor receptor	EGFR	31	0.01855442
Pro-epidermal growth factor	EGF	31	0.01146838
Heat shock protein HSP 90-alpha	HSP90AA1	30	0.01797037
Interleukin-10	IL10	30	0.01289718
Myc proto-oncogene protein	MYC	30	0.01966252
Proto-oncogene c-Fos	FOS	29	0.02549511
Nitric oxide synthase, endothelial	NOS3	29	0.02981536
Androgen receptor	AR	28	0.02001421
Transcription factor p65	RELA	28	0.00843228
Matrix metallopeptidase −2	MMP2	28	0.00490791
Heme oxygenase 1	HMOX1	28	0.01262935
Caveolin	CAV1	27	0.04377675
Caspase-8	CASP8	26	0.00536277
Hypoxia-inducible factor 1	HIF1A	26	0.00382947
Tumor necrosis factor receptor superfamily member 1A	TNFRSF1A	23	0.0025177
Signal transducer and activator of transcription 1	STAT1	23	0.00513282
Superoxide dismutase 1	SOD1	21	0.00477781
Cyclin-dependent kinase 4	CDK4	20	0.00416541
Matrix metalloproteinase 1	MMP1	19	0.00321129
Endoplasmic reticulum chaperone BiP	HSPA5	18	0.00332267
Protein kinase C	PRKCA	15	0.00150001

### 3.5. Analysis of KEGG pathways and GO biological processes

**Figure 5.**
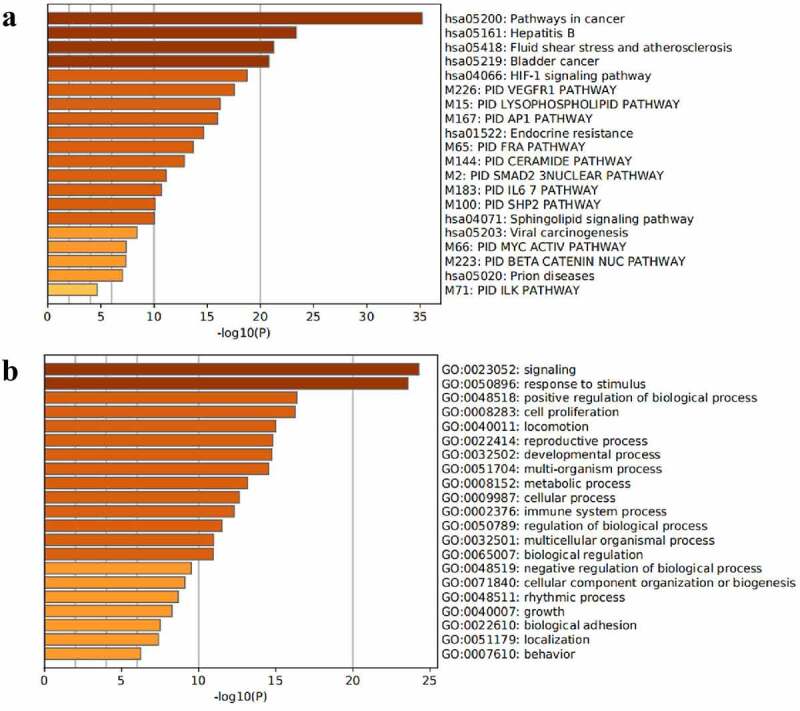
KEGG pathway and GO biological process enrichment analysis diagrams.

**Figure 6.**
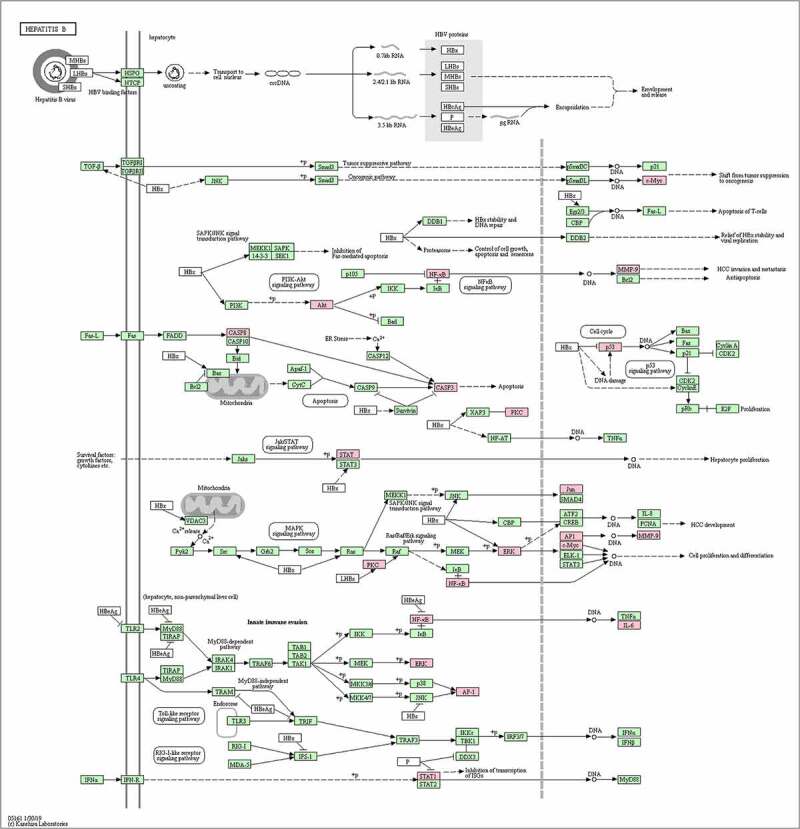
Annotated map of the target points of the main active components of *S. marianum* on NAFLD-related signal pathways.

A total of 30 core gene targets were finally obtained by KEGG pathway and GO analysis annotation. The *P* < 0.05 principle was used to filter the pathways, and the top 20 KEGG pathways were listed in order of *P*-value (Figure 5(a)). It is mostly concerned with Hepatitis B, cancer pathways, the IL pathway, the AP1 signaling pathway, the HIF-1 signaling system, the VEGFR1 pathway, prion disorders, endocrine resistance, the Myc pathway, the SMAD2 pathway, the SHP2 pathway, the FRA pathway, and so on. NAFLD is linked to several pathways, including the cancer pathway, the Hepatitis B pathway, the VEGFR1 signaling pathway, the IL-6 signaling pathway, the AP1 signaling pathway, and the HIF signaling pathway. The analysis results of the top 20 targets in GO biological processes are shown in Figure 5(b). These targets are associated with a wide range of biological processes such as biological adhesion, growth, rhythmic process, negative regulation of the biological process, biological regulation, regulation of the biological process, cellular component organization or biogenesis, multicellular organismal process, immune system process, cellular process, multi-organism process, metabolic process, developmental process, locomotion, reproductive process, cell proliferation, positive regulation of the biological process. The results showed that NAFLD is caused by a number of biological processes, and *S. marianum* may be hepatoprotective against NAFLD by regulating these biological processes. Annotated map of the target locations of active components of *S. marianum* in NAFLD-related pathways is shown in Figure 6.

### 3.6. Molecular docking analysis

**Figure 7.**
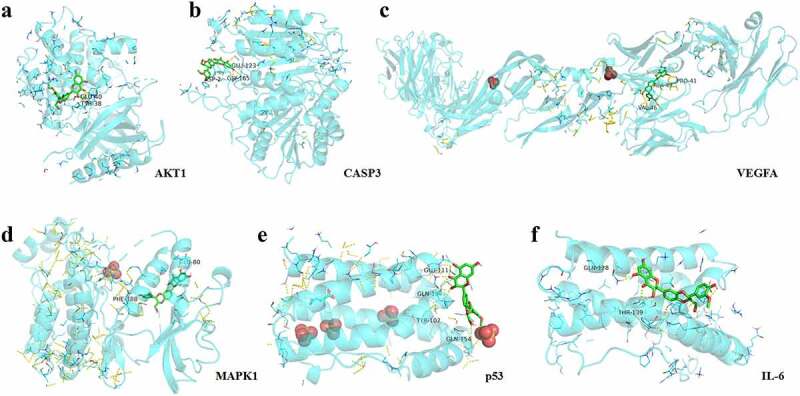
Docking pattern of silymarin with the key target molecules.

The molecular docking of silymarin with the above key targets has an average docking affinity of −6.77 kcal/mol (Table 4). The detailed docking with each key target is shown in Figure 7.

**Table 4.** The results of molecular docking

Compound	Target	PDB	Energy (kcal/mol)
Silymarin	AKT1	7NH5	−6.5
Silymarin	IL-6	1IL6	−6.5
Silymarin	CASP3	1CP3	−6
Silymarin	MAPK1	1PME	−7.2
Silymarin	p53	1AlU	−7.2
Silymarin	VEGFA	1BJ1	−7.2

### 3.7. Effects of silymarin on mice body weights and histopathological changes

**Figure 8.**
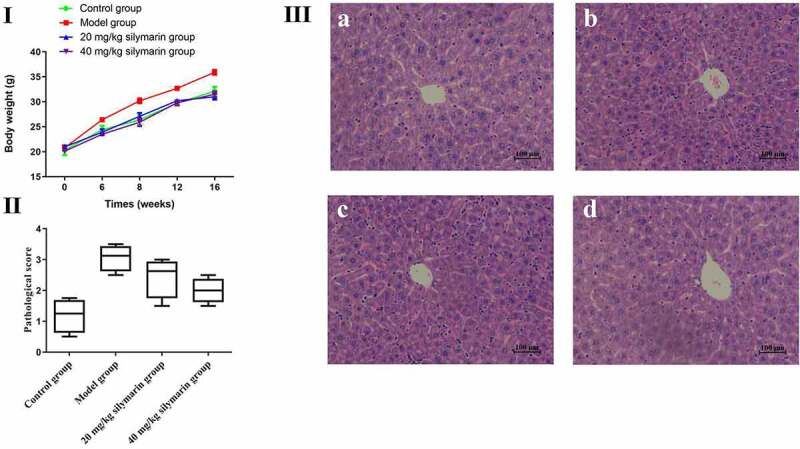
Effects of silymarin on mice body weights and histopathological changes.

Effects of silymarin on mice’s body weight are shown **in** Figure 8(a). In the present study, the mice’s body weight was significantly increased in the model group while gradually increasing in 20 mg/kg or 40 mg/kg silymarin groups, respectively (*P* < 0.01). The pathological score provided visual evidence related to the effect of silymarin on NAFLD. It was shown that silymarin exhibit the obviously hepatoprotective effect on NAFLD (Figure 8(b)). The effects of silymarin on histopathological changes are presented in Figure 8(c). In the control group, the structure of hepatocytes in liver tissue at different times was complete and clear, the structure of hepatic lobules was normal, and hepatocytes were arranged into the hepatic cord (Figure 8(c)). Compared with the control group, the hepatocytes were disordered, the nuclei were damaged in varying degrees, and the arrangement was irregular, with a certain degree of inflammation in the model group (Figure 8(c)). However, the pathological changes were significantly reversed by 20 mg/kg silymarin (Figure 8(c)) and 40 mg/kg silymarin treatment (Figure 8(d)). All the above results suggest that silymarin exhibit a significant hepatoprotective effect on NAFLD.

### 3.8. Blood lipid and liver function level

**Figure 9.**
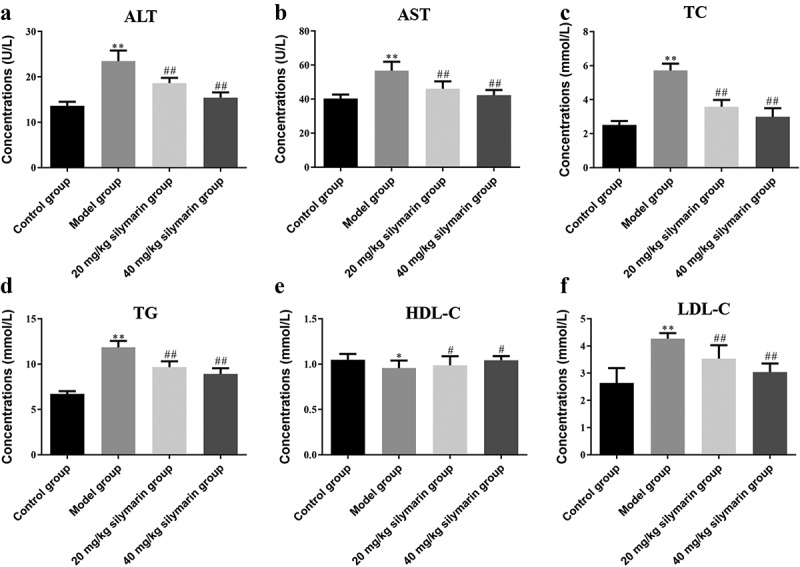
Effects of silymarin on blood lipid and liver function level.

Compared with the control group, the contents of ALT (Figure 9(a)) and AST (Figure 9(b)) in the serum of the model group were substantially higher than those of the control group (*P* < 0.01). In addition, the content of TC (Figure 9(c)) and TG (Figure 9(d)) were also significantly increased (*P* < 0.01), as well as the content of HDL-C (Figure 9(e)) and LDL-C (Figure 9(f)) significantly increased (*P* < 0.05) in the model group. It was revealed that the content of blood lipid in the model group increased, liver function was damaged to a certain extent, and obvious inflammatory lesions appeared. On the contrary, mice pretreated with silymarin showed a significant reduction in the level of these parameters (Figure 9).

### 3.9. Effects of silymarin on the expression of NAFLD-related proteins

**Figure 10.**
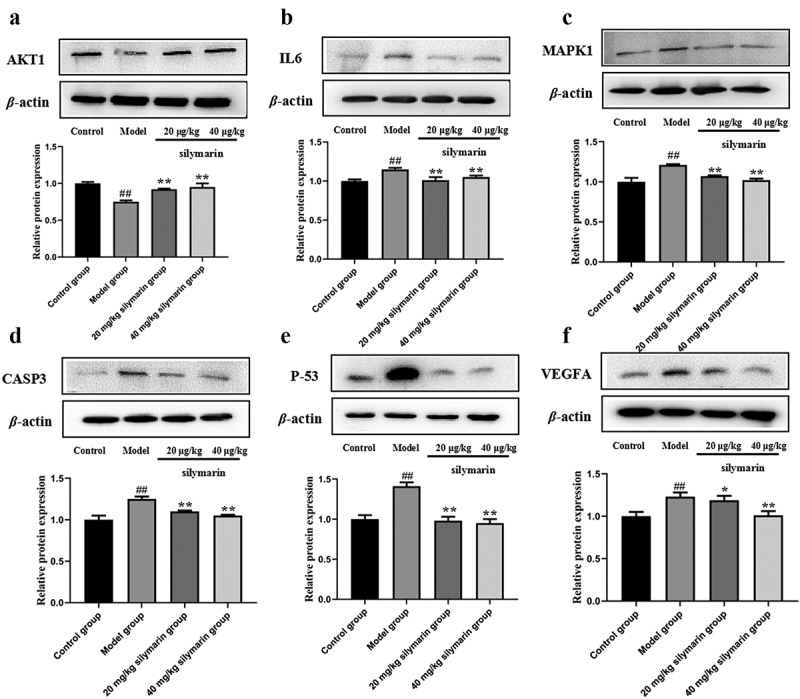
Effects of silymarin on the expression of related proteins of NAFLD.

To explore the effect of silymarin on NAFLD, relevant signaling pathways proteins (AKT1, IL-6, MAPK1, Caspase 3, p53, VEGFA) predicted by network pharmacology were investigated by Western blot (Figure 10). In the model group, AKT1 protein expression was lower than in the control group (Figure 10(a)), while IL-6, MAPK1, Caspase 3, p53, and VEGFA protein expression was higher (Figure 10(d-f)). In contrast, the low-dose silymarin group and the high-dose silymarin group had a tendency to up-regulate the AKT1 protein and down-regulate the IL-6, MAPK1, Caspase 3, p53, and VEGFA proteins (Figure 10(a-f)). These findings suggested that silymarin may have protective effects on NAFLD protection.

## 4. Discussion

In recent years, more and more related studies and researches are focused on conventional pharmacological strategies. Despite the pharmacology and mechanism of the drug was revealed by traditional pharmacological methods, it is difficult to explain the complicated interaction and molecular process that exists between medications and the human body [32]. Furthermore, the action of a single chemical medicine is limited to a single target, which lacks the benefits of overall demonstration as compared to TCM’ s and its compounds’ multi-component and multi-target synergistic impact. Network pharmacology, which is based on the advancements of systems biology and multi-directional pharmacology, integrates biological networks and drug action, analyzes the relationship between drugs and nodes or network modules in the network, and employs the action mode of multi-component and multi-target. In the current work, the TCMSP database was used to screen 11 active compounds, the DisGeNET database was used to find 1,058 gene targets linked to NAFLD, and Venny 2.1 was used to test 92 common targets. After that, the UniProt database was used to batch convert the abbreviated gene names, and the String database was utilized to search, predict, and build the PPI network. The topological properties of the PPI network were studied, and the PPI network diagram was drawn. The possible material basis and molecular mechanism of *S. marianum* on NAFLD were preliminarily analyzed by network pharmacology. The study is the first to propose a comprehensive strategy combining pharmacological experiments and network pharmacology methods to explore the material basis of *S. marianum* pharmacodynamics and its possible liver protection mechanism against NAFLD.

The liver is a dynamic organ that is involved in a variety of physiological activities, including the regulation of glucose and lipid metabolism throughout the body [33]. During the past 20 years, the prevalence of NAFLD has more than doubled, while the prevalence of other chronic liver diseases has remained consistent or even reduced [34]. Flavonoids are chemicals with the 2-phenylchromone structure that exhibit a variety of actions, including anticancer, antioxidant, and hepatoprotective effects on NAFLD [35]. Silymarin is a flavonoid that has been shown to maintain liver cell membranes, facilitate the fat transfer, and drastically improve liver enzyme levels in patients with NAFLD [36]. Zhu et al. studied how *S. marianum* extract affected hepatic steatosis and oxidative stress in mice fed a high-fat diet. The results demonstrated that *S. marianum* extract effectively lowered mice body weight, fat mass, serum triglyceride, free fatty acid, glucose, insulin, and other biochemical indicators [37]. Quercetin is a flavonoid with antioxidant, anti-inflammatory, antiviral, and anticancer properties. The effects of quercetin on insulin resistance and liver fat buildup in NAFLD were observed at the cellular level. The findings revealed that lipid accumulation and triglyceride levels rose considerably in HepG2 cells [38]. It was found that quercetin significantly improved the early NAFLD status of rats by controlling fatty acid-related metabolites (adrenaline, etc.), and regulating inflammation-related metabolites [39]. Vitamin E is the active ingredient of *S. marianum*, with antioxidant, anti-inflammatory, and anti-apoptotic properties in the treatment and prevention of NAFLD and NASH. Perumpail et al. have found that the usage of Vitamin E reduced liver enzymes while improving biochemical properties. Furthermore, the histological investigation revealed that Vitamin E administration improved lobular inflammation and hepatic steatosis [40].

KEGG pathway enrichment and GO biological process analysis were done on the proteins appearing in the PPI network to further investigate the biological pathways of NAFLD that are regulated by the active components of *S. marianum*. The major targets of the ‘*S. marianum*-NAFLD’ PPI network have been linked to cancer, hepatitis B and C, endocrine resistance, insulin resistance, type 2 diabetes, inflammatory bowel disease, and other disorders, according to KEGG pathway enrichment data. NAFLD refers to a group of progressive liver illnesses that include simple steatosis, NASH, fibrosis, and the possibility of evolving into cirrhosis and hepatocellular carcinoma. The majority of NAFLD patients develop liver cirrhosis, and the risk of liver cancer in NAFLD patients is higher than in the general clinical population [41]. It has been proven that hepatitis B metabolic variables play an essential role in the incidence of NAFLD patients, with type 2 diabetes being more severe [42]. It has been shown that type 2 diabetes is closely associated with NAFLD, and that NAFLD affects more than three-quarters of diabetic patients [43]. It was discovered that NAFLD could be diagnosed in 33.6% of patients with inflammatory bowel disease (IBD) in the lack of metabolic risk factors, proving that NAFLD in patients with IBD differs from NAFLD in the absence of IBD. The clinical history of tract disease leads to distinct NAFLD phenotypes, and more severe IBD appears to be associated with more severe steatosis [44]. Furthermore, the NAFLD-protective activity of *S. marianum* is linked to the IL6 pathway, the HIF-1 route, and the AP-1 pathway, all of which are enriched in the KEGG pathway. Fang et al. investigated whether IL-6/STAT3-mediated hepatic autophagosome activation and hepatocyte oxygen consumption contribute to NAFLD hepatoprotection. Caffeine’s inability to ameliorate IL-6 and hepatocyte-specific STAT3 gene deletion NAFLD in mice suggests that the IL6/STAT3 pathway is required for caffeine’s hepatoprotective action in NAFLD [45]. Chen et al. found that hypoxia-induced HIF-overexpression worsens NAFLD progression by blocking fatty acid oxidation and increasing hepatic adipogenesis via PPAR, demonstrating that the HIF-1 pathway is directly associated with NAFLD [46]. Hasenfuss et al. discovered that the AP-1 protein regulates NAFLD. This distinct antagonistic control of PPAR by various AP-1 dimers occurs at the transcriptional level. Obesity, hepatic lipid metabolism, and the relationship between NAFLD are all controlled by AP-1. Furthermore, Fos-related antigen 1 (Fra-1) and Fos-related antigen 2 (Fra-2) prevent dietary NAFLD by blocking prostate PPAR signaling, demonstrating that hepatocyte-specific Fra-1 expression can successfully reverse established NAFLD and associated liver damage [47].

The experimental verification was carried out for expanded relative indicators in silymarin treatment of NAFLD based on network pharmacology analysis. The biochemical parameter evidence examined in this study is supported by histopathological studies. Histological study of mice liver treated with gavage nutritional solution revealed apparent characteristics such as inflammatory liver tissue and neutrophil infiltration. However, silymarin therapy greatly reduced inflammatory liver tissue and neutrophil infiltration. ALT is an enzyme found in abundance in the cytoplasm of liver cells. ALT levels in healthy people’s serum are typically low. When liver cells experience apoptosis and injury, the ALT level in the serum rises dramatically [48]. AST levels rise as liver disease progresses, possibly as a result of direct liver cell destruction and membrane leakage, while AST levels can revert to normal in people with compensated cirrhosis [49]. Hashemi et al. in NAFLD patients has demonstrated that silymarin was efficient in reducing ALT and AST levels in comparison to placebo treatment which is similar to our study [50]. TG represents the major form of storage and transport of fatty acids within cells and in the plasma of NAFLD [51]. TC refers to the sum of cholesterol contained in all lipoproteins in the blood that the liver is the main organ for synthesis and storage of it [52]. It was reported that the TC and TG were closely associated with NAFLD [53]. In the present study, silymarin may significantly reduce the level of TC and TG in NAFLD mice which is consistent with the previous study. HDL-C is mainly synthesized by the liver and small intestine, and it is the smallest type of lipoprotein. Its main function is to transport excessive cholesterol in extrahepatic tissues to the liver for metabolism to prevent excessive accumulation of cholesterol in these tissues [54]. LDL-C is the main lipoprotein in fasting plasma, accounting for about two-third of plasma lipoproteins, and is the main vehicle for transporting cholesterol to extrahepatic tissues [55]. It was revealed that HDL-C and LDL-C were the risk factors of NAFLD, which may cause abnormal liver function [56]. In our study, we have been demonstrated that silymarin could significantly decrease the levels of HDL-C and LDL-C in NAFLD mice. Molecular docking suggests that silymarin has a good binding effect with the core targets. To explore the effect of silymarin on NAFLD, related signaling pathways proteins, which predict with network pharmacology (AKT1, IL-6, MAPK1, Caspase 3, p53, VEGFA) were detected by Western blot. The results show that silymarin might be involved in the protective effects of NAFLD by up-regulating the protein expression of AKT1 and down-regulating the protein expressions of IL-6, MAPK1, Caspase 3, p53, and VEGFA. It finally demonstrated that that silymarin may ameliorate NAFLD mainly through the apoptosis pathway, the inflammatory pathway, the Hepatitis B-related pathway, and the cancer-related pathway.

NAFLD mainly includes nonspecific symptoms and signs such as fatigue, indigestion, liver pain, hepatosplenomegaly, and may have metabolic syndrome-related symptoms such as overweight and/or visceral obesity, increased fasting blood glucose, dyslipidemia, and hypertension [57,58]. Through GO biological process analysis of gene targets, we found that NAFLD was related to the biological adhesion, response to stimuli, growth, cell component organization or biogenesis, negative regulation of biological processes, biological regulation, immune system processes, metabolic processes, reproductive processes, cell proliferation, and biological processes. Previous studies have shown that NAFLD patients have irregular homeostasis pathways, nonparenchymal cells in the liver are stimulated by lipid antigens and adipokines, which are similar to the above biological process [59]. Immune substances released by the body can alter the expression of certain critical proteins and regulate lipid metabolism, hence influencing the pathological process of NAFLD [60]. Hence, it is relatively important to study the above biological processes related to NAFLD.

## 5. Conclusion

Taken together, the present study puts forward for the first time a comprehensive strategy that combines pharmacological experiments and network pharmacology methods to explore the material basis of *S. marianum*’s pharmacodynamics and its possible hepatoprotective mechanism for NAFLD. Through active ingredient screening, target prediction, PPI network construction, KEGG pathway, GO biological process analysis, and experimental verification, *S. marianum*’s hepatoprotective mechanism for NAFLD was clarified. It has been demonstrated that the active hepatoprotective ingredient of *S. marianum* was silymarin on NAFLD. It could significantly improve the levels of ALT, AST, TC,TG, HDL-C, LDL-C, decrease protein expressions of IL-6, MAPK1, Caspase 3, p53, VEGFA, and increase protein expression of AKT1. It provides data support for the development of active ingredients of *S. marianum* and study on the hepatoprotective mechanism on NAFLD, and propose new ideas for the systematic and comprehensive study of NAFLD at the cellular and molecular levels, respectively.

## Supplementary Material

Supplemental Material

## Data Availability

The raw data used to support the findings of this study are available from the corresponding author upon request.
