# Supplementary material for: Hepatoprotective mechanism of Silybum marianum on nonalcoholic fatty liver disease based on network pharmacology and experimental verification
Source: Bioengineered. 2022 Feb 16;13(3):5216–35. doi: 10.1080/21655979.2022.2037374 (PMC8974060; doi:10.1080/21655979.2022.2037374)
Supplement: Supplemental Material [file KBIE_A_2037374_SM4654.pdf]

# Hepatoprotective mechanism of *Silybum marianum* on non-alcoholic fatty liver disease based on network pharmacology and experimental verification

**Guoyan Jiang[1], Chunhong Sun[1], Xiaodong Wang[2], Jie Mei[3], Chen Li[4], Honghong Zhan[5], Yixuan Liao[5], Yongjun Zhu[6]*, Jingxin Mao[5,7*]**

[1] Department of Emergency, The Third Affiliated Hospital of Chongqing Medical University, Chongqing, China.

[2] Chongqing Medical and Pharmaceutical College, Chongqing, China.

[3] Stomatological Hospital of Chongqing Medical University, Chongqing China.

[4] Department of Biology, Chemistry, Pharmacy, Free University of Berlin, Berlin, Germany.

[5] College of Pharmaceutical Sciences, Southwest University, Chongqing, China.

[6] Department of Orthopedics, The Ninth People's Hospital of Chongqing, Chongqing, China.

[7] College of Basic Medical Science, Southwest University, Chongqing, China.

[*] Jingxin Mao and Yongjun Zhu contributed equally to this work.

[*] **Corresponding author**: Jingxin Mao, Associate Prof and Ph. D.

College of Pharmaceutical Sciences, Southwest University, Chongqing 400715, China.

College of Basic Medical Science, Southwest University, Chongqing 400715, China.

**Email**: mmm518@163.com or maomao1985@email.swu.edu.cn

## List of supporting information

Figure S1.The images of the original western blots of AKT1.

Figure S2. The images of the original western blots of IL6.

Figure S3. The images of the original western blots of MAPK1.

Figure S4. The images of the original western blots of CASP3.

Figure S5. The images of the original western blots of p53.

Figure S6. The images of the original western blots of VEGFA.

Figure S7. The images of the original western blots of $\beta$-actin.

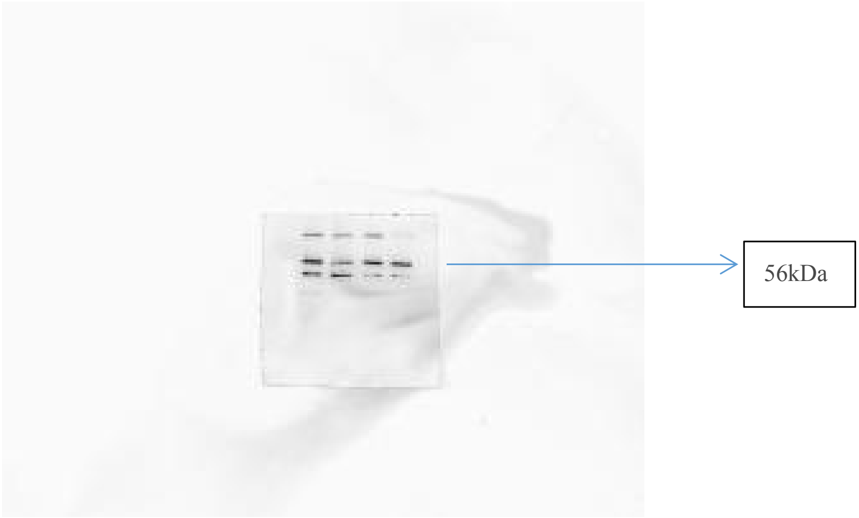


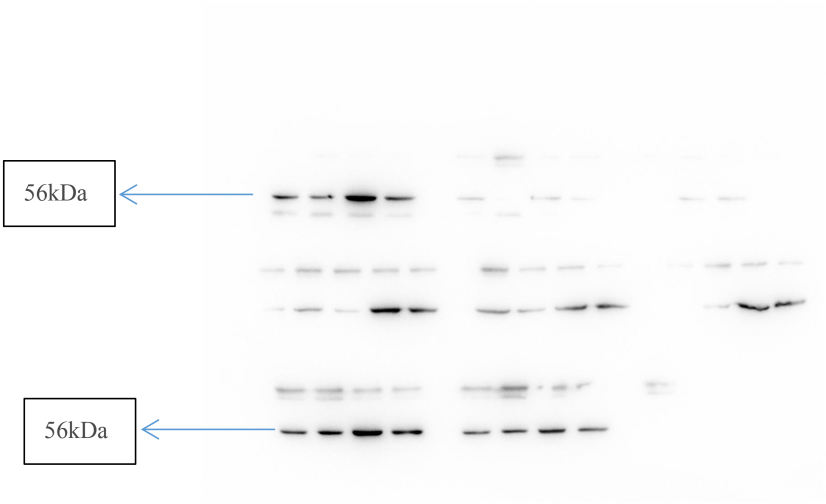


Figure S1.The images of the original western blots of AKT1.

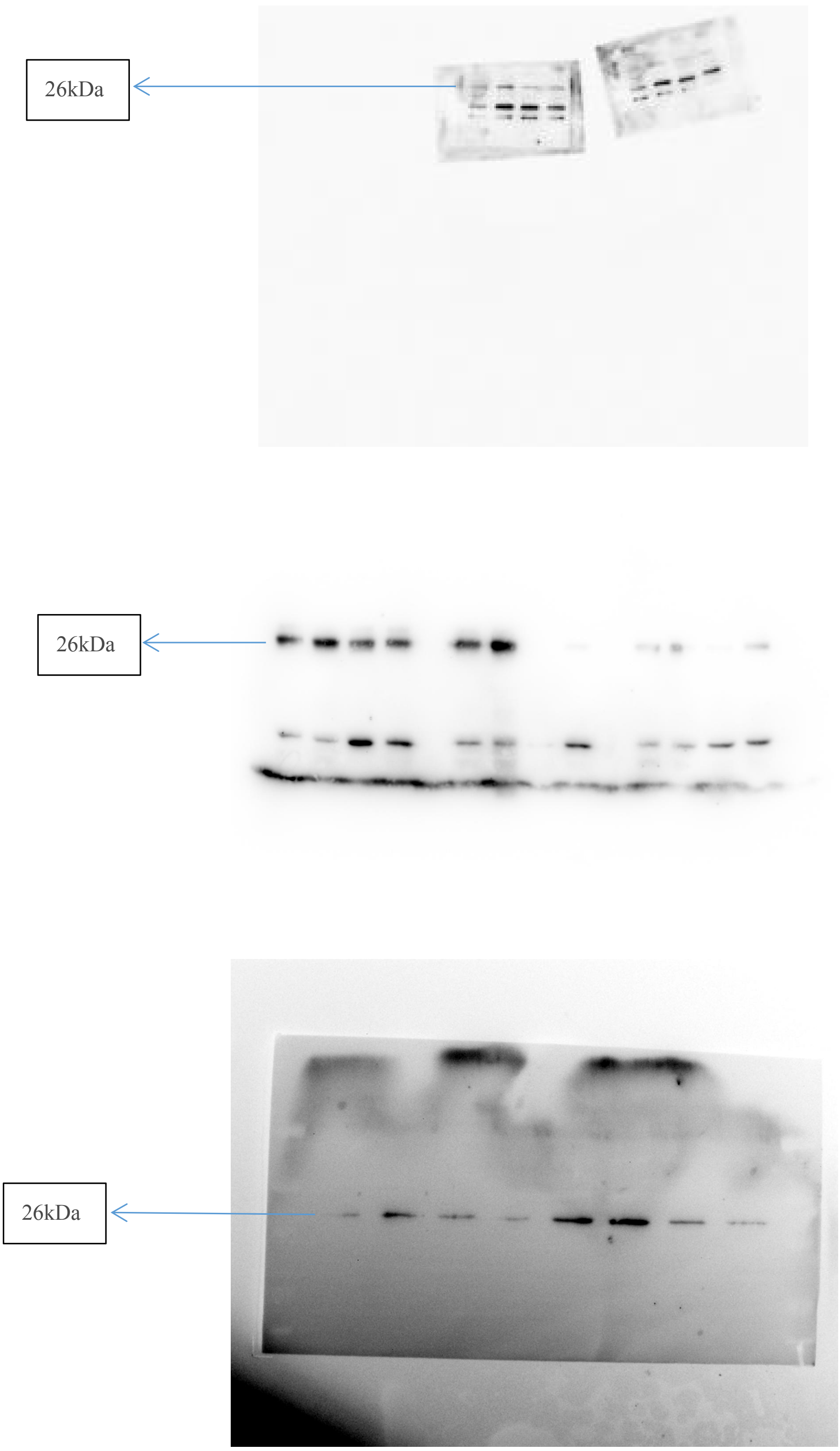


Figure S2. The images of the original western blots of IL6.

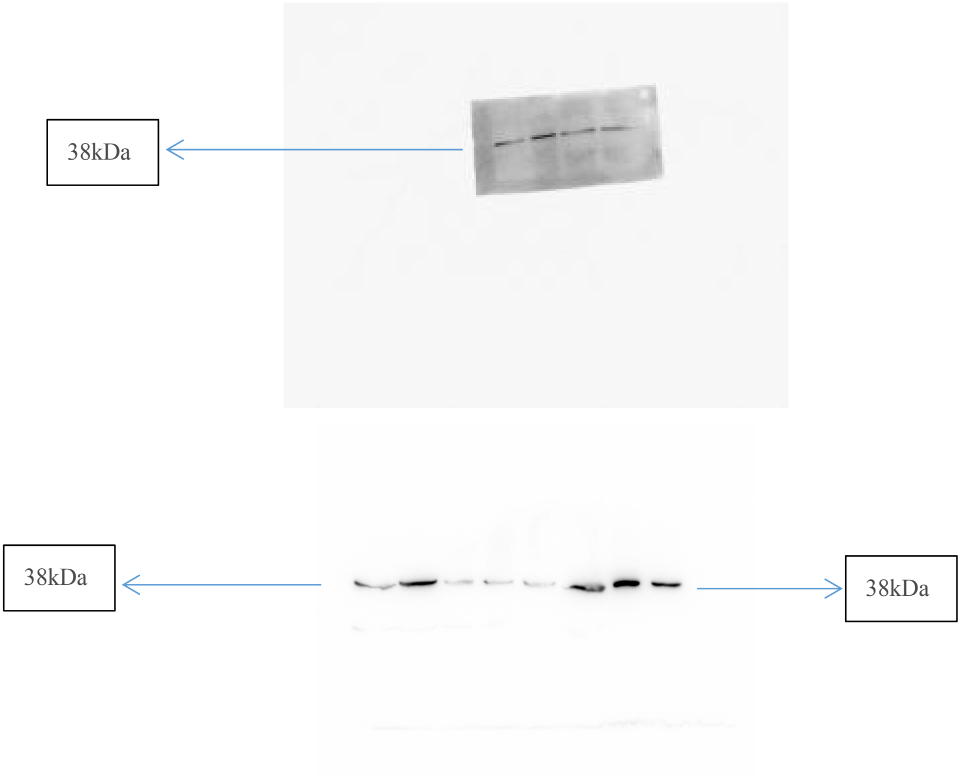


Figure S3. The images of the original western blots of MAPK1.

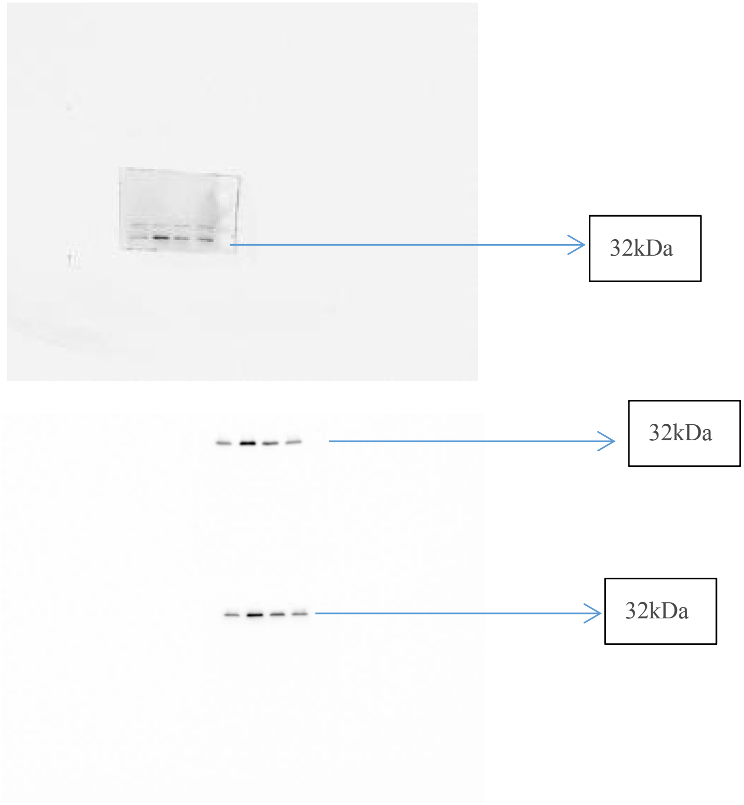


Figure S4. The images of the original western blots of CASP3.

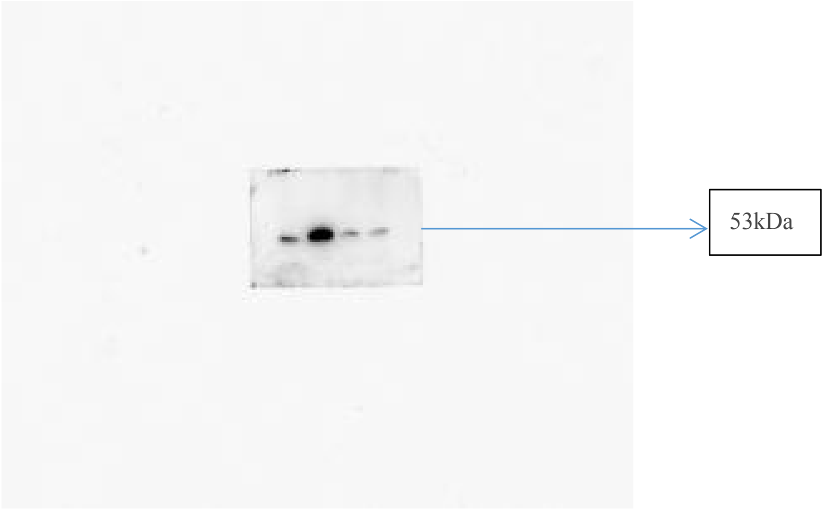


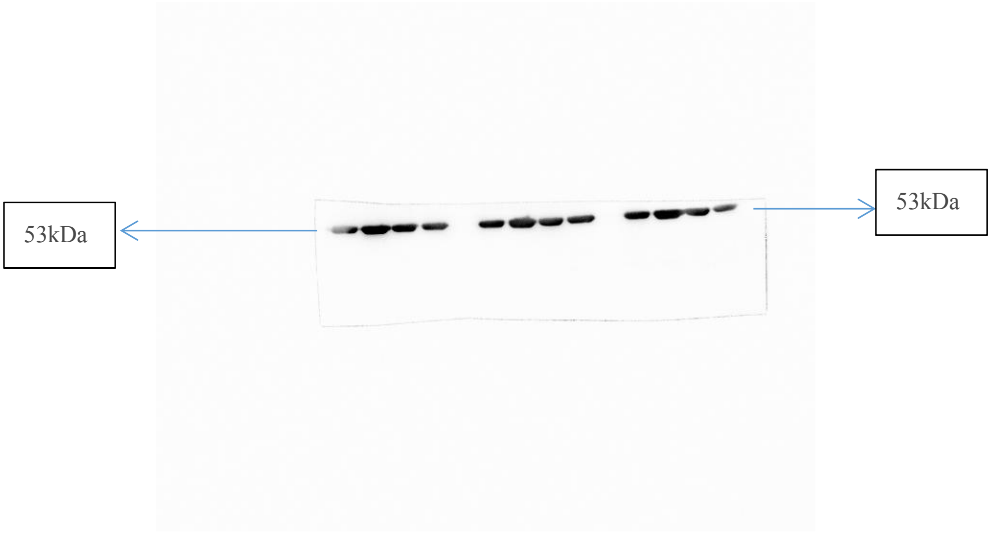


Figure S5. The images of the original western blots of p53.

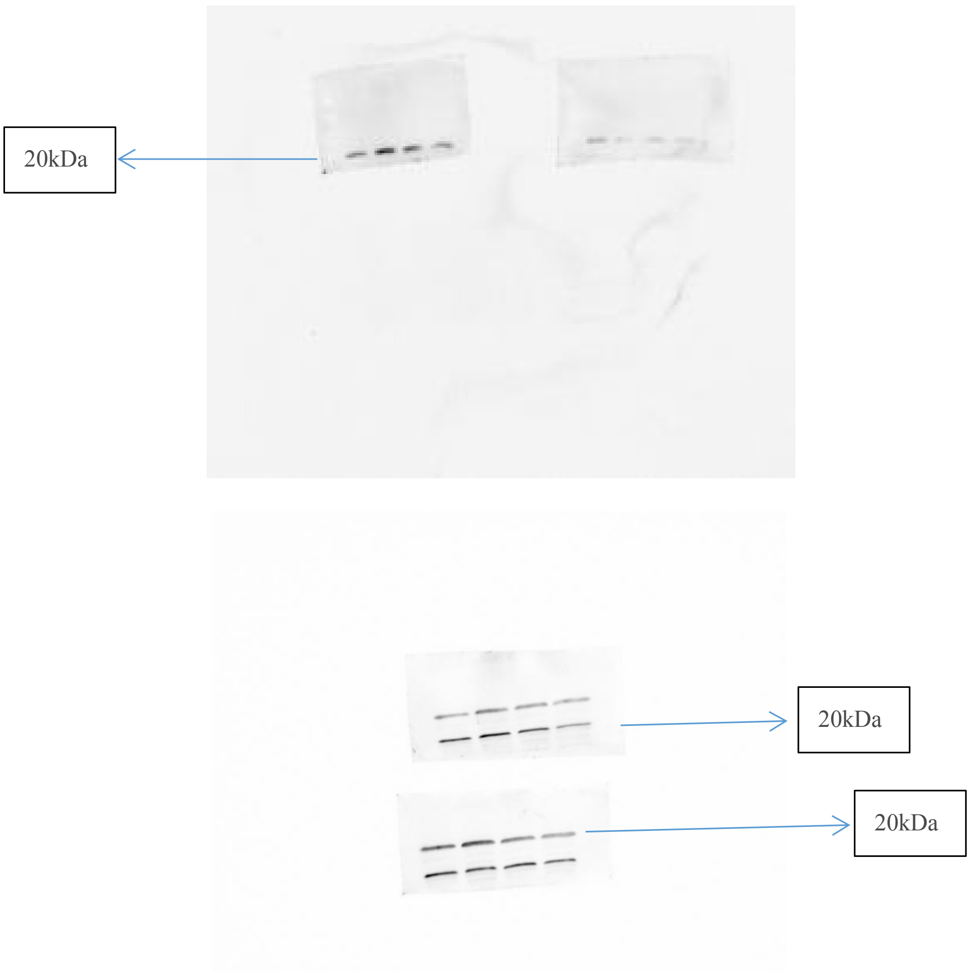


Figure S6. The images of the original western blots of VEGFA.

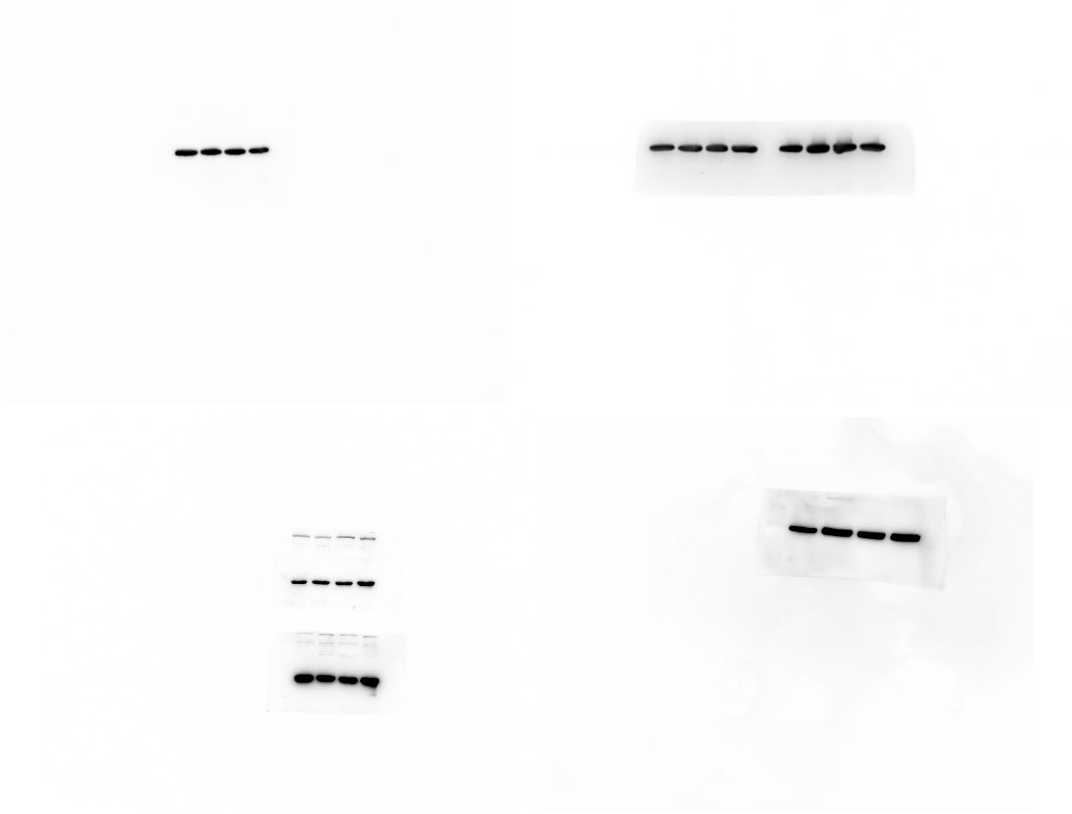

Figure S7. The images of the original western blots of $\beta$-actin.
